# Circulating immune cells in cerebral small vessel disease: a systematic review

**DOI:** 10.1007/s10522-025-10250-x

**Published:** 2025-05-05

**Authors:** L. Van der Taelen, A. M. Briones, T. Unger, J. Staals, R. J. van Oostenbrugge, S. Foulquier

**Affiliations:** 1 Department of Pharmacology and Toxicology, Maastricht University, Maastricht, The Netherlands; 2 Department of Neurology, Maastricht University Medical Center, Maastricht, The Netherlands; 3 CARIM – Research institute of Cardiovascular Diseases, Maastricht University, Maastricht, The Netherlands; 4 MHeNS – Research institute of Mental Health and Neuroscience, Maastricht University, Maastricht, The Netherlands; 5 Departamento de Farmacología, Facultad de Medicina, CiberCV, Universidad Autónoma de Madrid, Instituto de Investigación Hospital Universitario La Paz, Madrid, Spain; 6 Department of Pharmacology and Toxicology, Faculty of Health Medicine and Life Sciences, Maastricht University, Universiteitssingel 50, P.O. Box 616, 6200 MD Maastricht, The Netherlands

**Keywords:** Cerebral small vessel disease, Circulating immune cells, White matter hyperintensities, Lacunes, Microbleeds, Enlarged perivascular spaces

## Abstract

Cerebral small vessel disease (cSVD) refers to all pathologies of the brain’s arterioles, capillaries, and venules. cSVD is highly prevalent with ageing and is diagnosed by its characteristic neuro-imaging features. Emerging evidence suggests that circulating immune cells play an important role in cSVD’s pathology. However, the specific immune cell populations involved remain poorly understood. This systematic review synthesizes current evidence on circulating immune cells in cSVD and their associations with cSVD features. A systematic search was conducted using PubMed, Embase, and Web of Science databases. All studies investigating the association between peripheral immune cells and imaging features of cSVD were included. Data were extracted on study design, immune cells and cSVD measures, and outcomes. A total of 18 studies were included. Pro-inflammatory monocytes were associated with the severity and progression of cSVD over time. The neutrophil-to-lymphocyte ratio (NLR) showed positive associations with white matter hyperintensities (WMH) and enlarged perivascular spaces. The monocyte-to-HDL ratio (MHR) demonstrated a stronger association than the NLR with WMH, lacunes, and cerebral microbleeds. The lymphocyte-to-monocyte ratio (LMR) was linked to slower WMH progression and lower cSVD prevalence. Key findings highlight a role for pro-inflammatory circulating monocytes, NLR, MHR, and LMR in cSVD patients. These derived ratios serve as more reliable disease predictors than individual blood counts, showing potential as innovative diagnostic and prognostic markers. However, the reviewed studies predominantly employed cross-sectional and retrospective designs, suggesting the need for large-scale, prospective investigations to determine the role of these inflammatory markers in cSVD’s pathogenesis.

**Supplementary Information:**

The online version contains supplementary material available at 10.1007/s10522-025-10250-x.

## Introduction

Cerebral small vessel disease (cSVD) is an umbrella term that refers to all pathological processes that damage the small arteries, arterioles, venules, and capillaries in the brain (Pantoni 2010). It is considered an important cause of both ischemic and hemorrhagic stroke, and the most common pathology underlying vascular cognitive impairment (VCI) (Pantoni 2010). Most cSVD cases are related to age and vascular risk factors (Wardlaw et al. 2019; Cai et al. 2021). The presence of cSVD increases with age and as life expectancy continues to rise, the incidence of age-related cSVD increases as well, leading to a large social and economic burden (Cannistraro et al. 2019). Magnetic resonance imaging (MRI) features to determine the presence of cSVD include white matter hyperintensities (WMH), lacunes, cerebral microbleeds (CMB), and enlarged perivascular spaces (ePVS) (Wardlaw et al. 2013). Novel imaging features that might correspond better with clinical deficits, but are not in the clinical standard yet, are diffusion tensor imaging-derived peak width of skeletonized mean diffusivity (PSMD) and free water (Duering et al. 2023). Deep medullary veins (DMVs) can be assessed using susceptibility-weighted imaging (SWI) (Xu et al. 2020). The progression of imaging characteristics of cSVD is typically gradual over years but accelerates during aging and in the presence of classic vascular risk factors (Noz et al. 2018). Despite the increase in cSVD burden, the exact pathophysiological mechanisms are still poorly understood. Therapeutic options are therefore limited, with risk factor management remaining the mainstay of cSVD prevention and treatment (Whittaker et al. 2022).

Recent studies have highlighted a role for inflammation and immune cells in the development of cSVD (Wardlaw et al. 2019). The supposed mechanism is that immune cells acquire a pro-inflammatory phenotype that can lead to endothelial activation, neurovascular unit dysfunction, increased blood–brain barrier (BBB) permeability and subsequent brain lesions, ultimately resulting in impaired cognitive function (Bailey et al. 2012; Rouhl et al. 2012b). Despite increasing research interest, comprehensive reviews that synthesize existing findings on immune cell composition and its associations with cSVD imaging markers are still lacking. As a result, our understanding of the precise role of the immune system—and the key cellular players involved—in the development and progression of cSVD remains limited.

Therefore, in this study, we aim to systematically examine the current body of evidence to provide a comprehensive overview of immune cell compositions in cSVD patients and their associations with cSVD imaging markers. Furthermore, this review critically evaluates the quality and gaps in the existing literature and provides recommendations for future research.

## Methods

### Search strategy

We conducted a systematic search to identify studies reporting the association of circulating immune cells and cSVD. Publications were identified through PubMed, Web of Science and Embase search engines, and reported following the Preferred Reporting Items for Systematic review and Meta-Analysis Protocols (PRISMA-P). The search was completed on August 5, 2024. The search strategy is shown in Table 1.

**Table 1:** Search strategy

Search engine	Search terms
PubMed	("Cerebral Small Vessel Diseases"[MeSH Terms:noexp] OR "cerebral small vessel disease*"[Title/Abstract] OR "CSVD"[Title/Abstract]) AND ("Leukocytes"[MeSH Terms] OR "leukocyte*"[Title/Abstract]) NOT (Review[Publication Type])
Web of Science	TS = (“cerebral small vessel disease*” or CSVD) AND TS = (leukocyte*) NOT DT = (review)
Embase	('cerebral small vessel disease*' or CSVD).ti,ab,kf. and (exp leukocyte/ or leukocyte*.ti,ab,kf.) not review.pt

### Inclusion and exclusion criteria

The titles and abstracts identified by this search strategy were screened for relevance and eligibility. Full texts of relevant articles were read and selected according to the eligibility criteria. Only full-length, original research papers in English were considered for inclusion. All eligible articles were required to include at least one immune cell marker, and at least one cSVD MRI marker (WMH, CMB, ePVS). Studies involving non-sporadic cSVD and non-human studies were excluded.

### Data extraction

The following data was extracted from each included study: first author, year of publication, study population/design, measure of cSVD and circulating immune cells, and results. Principle findings of all studies are summarized in Table 2.

**Table 2:** Summary of studies

Authors (year)	Study design/population	Measure of cSVD	Circulating immune cells/inflammatory markers	Results
Cai et al. (2021)	Retrospective cohort study with 55 acSVD patients and 62 healthy controls	WMH Lacunes BG-ePVS CMB	WBC count Neutrophil count	Majority of WBC were neutrophils ↑ Pro-inflammatory factors in WBC of acSVD patients
Cai et al. (2024)	Retrospective, observational study with 368 patients with cSVD on neuro-imaging and 299 non-cSVD	WMH Lacunes ePVS	WBC count Neutrophil count Lymphocyte count NLR	No difference in WBC count between groups ↑ Neutrophil count in cSVD patients ↑ NLR in cSVD patients ↓ Lymphocyte count in cSVD patients NLR is associated with moderate to severe WMH, PWMH, DWMH, EPVS, EPVS-BG, EPVS-CSO but not lacunes
Chung et al. (2020)	Retrospective, cross-sectional observational study of 950 neurologically healthy participants	WMH Lacunes	Neutrophil count Lymphocyte count NLR	NLR is not associated with lacunes and WMH Higher prevalences of lacunes and WMHs in the highest NLR group
Hou et al. (2022)	Retrospective, cross-sectional observational study with 66 cSVD patients with CI and 81 cSVD patients without CI	WMH Lacunes ePVS CMB	Neutrophil count Lymphocyte count Monocyte count NLR	No difference in neutrophil count between groups No difference in lymphocyte count between groups ↑ NLR in cSVD patients with CI NLR is associated with CI in cSVD patients
Jiang et al. (2022)	Cross-sectional observational study of 3052 community-dwelling residents	WMH Lacunes CMB BG-ePVS	WBC count Neutrophil count Lymphocyte count NLR	↑ WBC count in participants with cSVD markers ↑ Neutrophil count in participants with cSVD markers No difference in lymphocyte count between groups ↑ NLR in participants with cSVD markers Positive correlation between higher neutrophil count and lacune, and moderate-to-severe BG-ePVS No association between neutrophil count and WMH, CMBs, and severe BG-ePVS No association between NLR and WMH, lacunes, CMBs, severe BG-ePVS
Li et al. (2023)	Retrospective, single-center study with 288 cSVD patients and 299 non-cSVD	WMH Lacunes ePVS CMB	MHR WBC count Monocyte count Neutrophil-to-HDL ratio LMR Lymphocyte count Neutrophil count	↑ WBC count in cSVD patients ↑ Monocyte count in cSVD patients ↑ MHR in cSVD patients ↑ Neutrophil-to-HDL ratio in cSVD patients ↓ LMR in cSVD patients No difference in neutrophil count and lymphocyte count between groups MHR is not associated with the risk of cSVD
Nam et al. (2017)	Retrospective, single-center cohort study with 2875 healthy subjects	WMH Lacunes CMB	WBC count Neutrophil count Lymphocyte count NLR	WBC count, neutrophil count, and NLR are associated with WMH volume Lymphocyte count is not associated with WMH volume
Nam et al. (2024)	Retrospective cross-sectional observational study with 3144 healthy participants	WMH Lacunes CMB	WBC count Neutrophil count Lymphocyte count Monocyte count MHR NLR	No difference in WBC and lymphocyte count between patients with lacunes and without ↑ Neutrophil, monocyte count, NLR, and MHR in patients with lacunes vs without No difference in neutrophil, lymphocyte counts and NLR between patients with CMBs and without ↑ WBC, monocyte counts, and MHR in patients with CMBs vs without WBC, neutrophil, monocyte counts, NLR, and MHR are associated with WMH volume, lacunes, and CMBs NLR is associated with WMH volume but not lacunes and CMBs Lymphocyte counts are not associated with WMH volume
Noz et al. (2018)	Prospective cohort study with cross-sectional component with 51 cSVD patients and no control group	WMH	WBC count Monocyte count Classical monocytes (%) Intermediate monocytes (%) Non-classical monocytes (%)	WBC count is not associated with WMH Total monocyte count is not associated with WMH volume Classical and non-classical monocytes are not associated with WMH Intermediate monocytes are associated with WMH
Noz et al. (2021)	Longitudinal observational study with 35 participants of the RUN DMC-InTENse cohort	WMH Lacunes CMB	WBC count Neutrophil count Lymphocyte count Monocyte count Classical monocytes (%) Intermediate monocytes (%) Non-classical monocytes (%)	WBC count, neutrophil count, lymphocyte count, total monocyte count and %, classical monocytes (%), intermediate monocytes (%), non-classical monocytes (%) are not different in participants with cSVD progression and without Pro-inflammatory monocyte phenotype is associated with progression of cSVD
Oberheiden et al. (2010)	Case–control study with 24 cSVD patients and 10 controls	WMH	Monocyte activation	cSVD patients have monocyte activation but no correlation between severity of cSVD and the degree of monocyte activation
Rouhl et al. (2012a)	Cross-sectional cohort study with 32 hypertensive patients with cSVD and 29 hypertensive patients without cSVD	WMH Lacunes CMB	Angiogenic T cells count T cell count	↓ T cell count in hypertensive patients with cSVD ↓ Angiogenic T cell count in hypertensive patients with cSVD ↑ Angiogenic T cell numbers are related to absence of cSVD ↑ T cell numbers are related to absence of cSVD
Tian et al. (2023)	Cross-sectional cohort study with 1909 community-dwelling participants	WMH Lacunes ePVS-BG, ePVS-CSO CMB DMVs	WBC count Neutrophil count Lymphocyte count Monocyte count	↓ WBC count in high DMV scores group ↓ Neutrophil count in high DMV scores group ↓ Lymphocyte count in high DMV scores group ↓ Monocyte count in high DMV scores group WBC and lymphocyte counts are related to DMV scores No association between neutrophil count and DMV scores No association between monocyte count and DMV scores
Wang et al. (2022)	Cohort study with 466 cSVD patients and 413 controls	WMH Lacunes ePVS CMB	WBC count Neutrophil count NLR	↑ NLR in cSVD patients No difference in WBC count, neutrophil count between cSVD patients and control NLR is associated with PWMH and DWMH NLR is related to EPVS
Xiao et al. (2024)	Longitudinal study with participants from Alzheimer’s Disease Neuroimaging Initiative (ADNI) database without Alzheimer’s Disease	WMH Lacunes CMB	Lymphocyte count Monocyte count Neutrophil count LMR NLR	↓ Lymphocyte count in Fazekas grade 1–3 compared to Fazekas grade 0 ↑ Monocyte count in Fazekas grade 1–3 compared to Fazekas grade 0 ↑ Neutrophil count in Fazekas grade 1–3 compared to Fazekas grade 0 ↑ Neutrophil count in the presence of CMB No difference in lymphocyte count, monocyte count, LMR, and NLR in the presence of CMB vs without No difference in lymphocyte count, monocyte count, neutrophil count, LMR, and NLR in the presence of lacunes vs without ↓ LMR in individuals with higher WMH grades ↑ NLR in individuals with higher WMH grades Lymphocytes, NLR are not associated with WMH volume Monocytes, neutrophils, LMR are associated with WMH volume Lymphocytes, monocytes are not associated with WMH volume change rate Neutrophils, LMR, and NLR are associated with WMH volume change rate A greater LMR at baseline is associated with slower WMH volume progression and lower scores of DWMH and PVMH No association between WMH volume and lymphocytes or NLR No association between lymphocyte count, monocyte count, neutrophil count, LMR, and NLR and CMBs or lacunes
Yu et al. (2023)	Prospective single-centered study with 32 acSVD patients and 28 controls	WMH ePVS CMB Lacunes	NK cell count WBC count Neutrophil count Lymphocyte count Monocyte count Eosinophil count Basophilic granulocyte count	↑ NK cells in WMH burden 2–3 No difference in WBC count, neutrophil count, lymphocyte count, and monocyte count between WMH burden 0–1 and WMH burden 2–3
Zhang et al. (2022)	Prospective observational study with 305 ICH patients	WMH ePVS CMBs Lacunes Small DWI lesions	WBC count Neutrophil count	No difference in WBC and neutrophil count between individuals with DWI lesions and without
Zhu et al. (2019)	Retrospective study with 87 cSVD patients and 30 non-cSVD controls	WMH	Leukocyte count Neutrophil count	No difference in leukocyte and neutrophil count between cSVD patients and healthy controls

*cSVD* cerebral small vessel disease, *acSVD* arteriosclerotic cSVD, *WMH* white matter hyperintensities, *PWMH* perivascular white matter hyperintensities, *DWMH* deep white matter hyperintensities, *DMV* deep medullary veins, *ePVS* enlarged perivascular spaces, *BG* basal ganglia, *CSO* centrum semiovale, *acSVD* arteriosclerotic cSVD, *CMB* cerebral microbleeds, *WBC* white blood cell, *NK cell* natural killer cell, *NLR* neutrophil-to-lymphocyte ratio, *LMR* lymphocyte-to-monocyte ratio, *MHR* monocyte-to-HDL ratio, *ICH* intracerebral hemorrhage, *CI* cognitive impairment

## Results

### Literature search and study characteristics

The entire literature search identified 79 unique articles, of which 60 were excluded based on their title/abstract. Searching the reference lists of relevant articles identified four additional studies meeting our inclusion criteria, resulting in 23 articles eligible for full text review. Five articles were removed after full text review, resulting in 18 final articles which were included in this systematic review. The eligibility screening process and reasons for exclusion are detailed in the flowchart provided (Suppl. Fig. 1).

### Circulating immune cells in cerebral small vessel disease

#### White blood cells

Multiple studies have investigated white blood cell (WBC) count in cSVD patients and individuals with cSVD imaging markers (Zhu et al. 2019; Noz et al. 2021; Jiang et al. 2022; Wang et al. 2022; Li et al. 2023; Cai et al. 2024; Nam et al. 2024). While findings were inconsistent across studies, the majority of studies found no significant difference in WBC count between cSVD patients and controls or between participants with cSVD markers and controls (Zhu et al. 2019; Noz et al. 2021; Jiang et al. 2022; Wang et al. 2022; Li et al. 2023; Cai et al. 2024; Nam et al. 2024). Similarly, in a prospective study by Zhang et al. including patients with an intracerebral hemorrhage, no difference in WBC count was found in patients with or without small DWI-positive lesions (Zhang et al. 2022).

Additionally, Cai et al. explored arteriosclerotic cSVD (acSVD), a major subtype within the cSVD spectrum (Cai et al. 2021). Total acSVD burden was assessed according to an ordinal cSVD score (0 to 4) based on STRIVE criteria (Cai et al. 2021). They found that circulating WBCs, the majority of whom were neutrophils, of patients with acSVD expressed higher levels of chemokine receptors, adhesion molecules, and BBB damage-associated matrix metalloproteinase (Cai et al. 2021). Higher levels of chemokine receptors *CXCR1*, *CCR6*, *CCR7*, adhesion molecules *ICAM1* and *VCAM1*, and matrix metalloproteinases *MMP8* and *MMP9* were found in the circulating leukocytes of patients with acSVD, suggesting endothelial activation and BBB dysfunction (Cai et al. 2021).

Furthermore, the association of WBCs with imaging markers of cSVD has been investigated in two retrospective studies by Nam et al. (2017; 2024). They included healthy participants that were assessed for markers of cSVD and found that WBC counts are associated with WMH volume in univariate regression analysis in both studies (Nam et al. 2017, 2024). On the other hand, the prospective study by Noz et al. investigated the association of WBC count with WMH in 51 cSVD patients and found that WBC count is not associated with WMH volume (Noz et al. 2018). Other studies investigating associations with conventional neuro-imaging markers of cSVD were not found.

In addition to conventional imaging markers, Tian et al. conducted a cross-sectional study with community-dwelling participants to investigate the association of DMVs with cSVD, employing a semiquantitative DMV scoring method (Tian et al. 2023). Participants were divided into two groups according to the median DMV scores. A lower WBC number in the high DMVs scores group was found (Tian et al. 2023). Furthermore, they report that WBC counts are related to DMV scores (Tian et al. 2023). However, they did not identify a strong association between DMVs and total burden or traditional imaging markers of cSVD, implying that the pathogenic mechanisms of DMVs and typical imaging markers of cSVD may differ (Tian et al. 2023).

In conclusion, while WBC count was associated with WMH volume in health check-up participants, no such association was found in cSVD patients. However, the limited data available as well as the variability in study designs, patient populations, and MRI markers may contribute to the lack of consensus. These findings highlight the importance of investigating individual immune cell populations to provide a clearer understanding of their associations with cSVD.

#### Neutrophils

Given the inconsistent findings for total WBC counts, specific leukocyte subpopulations, including neutrophils, could provide a better understanding of cSVD’s pathology. Total neutrophil count showed inconsistent results between participants with cSVD markers or cSVD patients and controls (Zhu et al. 2019; Noz et al. 2021; Hou et al. 2022; Jiang et al. 2022; Wang et al. 2022; Zhang et al. 2022; Li et al. 2023; Yu et al. 2023; Cai et al. 2024; Nam et al. 2024; Xiao et al. 2024).

The association of neutrophil count with conventional imaging markers of cSVD was investigated in four studies that focused on participants with markers of cSVD (Nam et al. 2017, 2024; Jiang et al. 2022; Xiao et al. 2024). Jiang et al*.* conducted a retrospective study and found no association between neutrophil count and WMH volume (Jiang et al. 2022). In contrast, neutrophil count was positively associated with WMH volume in two other studies (Nam et al. 2017; 2024). These findings were supported by a longitudinal study in which adults without dementia were investigated for markers of cSVD (Xiao et al. 2024). They found that neutrophils are associated with WMH volume as well as WMH volume progression (Xiao et al. 2024).

Furthermore, Jiang et al*.* also reported a positive correlation between higher neutrophil count and lacunes and between higher neutrophil count and moderate-to-severe ePVS in basal ganglia (BG-ePVS) (Jiang et al. 2022).

However, no association was found between neutrophil count and CMBs, or between neutrophil count and severe BG-ePVS (Jiang et al. 2022).

In addition to conventional imaging markers, the study by Tian et al. reported a decrease in neutrophil count in high DMV scores group and no association between neutrophil count and DMV scores (Tian et al. 2023).

Taken together, the associations of neutrophil count with imaging markers of cSVD were primarily investigated in participants in whom conventional MRI markers were analyzed. The lack of consistent results and newer markers highlights the need for further research in this area.

#### Monocytes

Monocytes emerged as another critical inflammatory cell type in the pathogenesis of cSVD (Li et al. 2023). While most studies reported an increase in total monocyte count in cSVD patients or participants with cSVD markers, the results were overall inconsistent (Noz et al. 2021; Li et al. 2023; Yu et al. 2023; Nam et al. 2024; Xiao et al. 2024).

Moreover, associations were found between monocytes and conventional cSVD imaging markers (Noz et al. 2018; Nam et al. 2024; Xiao et al. 2024). Two studies investigated monocyte count and associations with cSVD markers in participants that were analyzed for MRI markers of cSVD. Both studies reported that monocyte counts are associated with WMH volume (Nam et al. 2024; Xiao et al. 2024). However, Xiao et al*.* furthermore reported that monocytes are not associated with the progression of WMH volume (Xiao et al. 2024). One prospective study investigated monocyte count and its association with imaging markers in 51 cSVD patients (Noz et al. 2018). They reported that total monocyte count is not associated with WMH volume (Noz et al. 2018).

To better understand their contributions, monocytes can be classified into three subsets: classical (CD14++CD16−), intermediate (CD14++CD16+), and non-classical (CD14+CD16++) monocytes (Noz et al. 2018, 2021). While classical and non-classical monocytes were not associated with WMH, Noz et al. reported a positive association between intermediate monocytes and WMH (Noz et al. 2018). Moreover, Noz et al. demonstrated in a follow-up study that the monocyte cytokine production capacity was associated with WMH development over a 9-year period in participants with markers of cSVD (Noz et al. 2021).

In the longitudinal study by Xiao et al*.*, monocytes were not associated with lacunes or CMBs in participants with markers of cSVD (Xiao et al. 2024).

In addition to conventional imaging markers, Tian et al. reported a decrease in monocyte count in high DMV scores groups and no association between monocyte count and DMV scores (Tian et al. 2023). Other studies investigating associations with conventional imaging markers were not found.

Furthermore, research has increasingly focused on specific activation markers that provide deeper insight into different monocyte functional states (Oberheiden et al. 2010; Rouhl et al. 2012a). Oberheiden et al*.* performed a case–control study with 24 cSVD patients and observed no correlation between cSVD severity and the degree of monocyte activation, even though all cSVD patients had a significant monocyte activation compared to healthy individuals (Oberheiden et al. 2010).

The role of monocytes in chronic inflammation has been further explored through their interaction with high-density lipoprotein (HDL) cholesterol (Nam et al. 2024). As a result, the monocyte-to-HDL ratio (MHR) has emerged as a potential marker of the inflammatory function of monocytes (Li et al. 2023; Nam et al. 2024). In the retrospective study by Li et al. with 288 cSVD patients and 299 non-cSVD, higher MHR was found in cSVD patients (Li et al. 2023). However, the MHR was not associated with the risk of cSVD (Li et al. 2023). In another retrospective study with healthy participants, the MHR was associated with WMH volume, lacunes, and CMBs (Nam et al. 2024).

In conclusion, the associations between total monocyte count and imaging markers of cSVD demonstrated inconsistent results. Different monocyte subsets and their activation markers may provide clearer insights and a better understanding of their role in cSVD.

#### Lymphocytes

Total lymphocyte count demonstrated primarily no significant difference between participants with cSVD markers or cSVD patients and controls (Noz et al. 2021; Hou et al. 2022; Jiang et al. 2022; Li et al. 2023; Yu et al. 2023; Cai et al. 2024; Nam et al. 2024; Xiao et al. 2024).

Moreover, in two retrospective studies with healthy subjects that were screened for markers of cSVD, lymphocyte count was not associated with WMH volume (Nam et al. 2017, 2024). Similarly, no association between lymphocyte count and WMH volume and progression was reported in the longitudinal study by Xiao et al*.* in individuals with cSVD markers (Xiao et al. 2024).

Moreover, lymphocyte count was not associated with CMB or lacunes in subjects with cSVD markers (Xiao et al. 2024).

In addition to conventional cSVD markers, Tian et al. reported a decrease in lymphocyte count in high DMV scores group and an association of lymphocyte counts with DMV scores (Tian et al. 2023). No other studies investigated lymphocyte count and its associations with conventional and new MRI markers.

The lack of associations between lymphocyte count and markers of cSVD may suggest that specific lymphocyte subpopulations, particularly T and B cell subtypes, could provide more nuanced insights.

More specifically, angiogenic T cells (T_ang_), characterized as CD3+/CD31+/CD184+ lymphocytes, may regulate endothelial progenitor cell (EPC) function and potentially attenuate endothelial dysfunction (Rouhl et al. 2012a). Rouhl et al. performed a study with hypertensive patients with cSVD and reported a decrease in T cell count and a decrease in angiogenic T cell count in hypertensive patients with cSVD compared to hypertensive patients without cSVD (Rouhl et al. 2012a). Moreover, higher T cell count and higher angiogenic T cell count were related to the absence of cSVD (Rouhl et al. 2012a).

#### Natural killer cells

Yu et al*.* conducted a prospective study with 32 acSVD patients and 28 controls (Yu et al. 2023). They reported increased NK cell numbers in acSVD patients with heavy WMHs (Yu et al. 2023). Furthermore, through in vitro coculture experiments of human neurons and NK cells, they demonstrated that NK cells highly express integrin β2 (ITGB2) and bind to ICAM1 on BBB endothelial cells via immune synapses, releasing cathepsin D (CTSD) and exacerbating BBB damage (Yu et al. 2023). Proteomic analysis revealed NK cell activation as indicated by the high expression of ITGB2, cathepsin D, filamin-A (FLNA), and granzyme H (Yu et al. 2023). Notably, ITGB2 overexpression enhanced NK cell binding to ICAM1 on BBB endothelial cells, while aberrant FLNA expression was associated with cell-extracellular matrix interactions (Yu et al. 2023).

Only one study reported NK counts in acSVD patients, requiring further research to draw definitive conclusions.

#### Neutrophil-to-lymphocyte ratio

Compared to individual blood indices, the neutrophil-to-lymphocyte ratio (NLR) emerged as a potentially better predictor of inflammatory diseases (Chung et al. 2020; Cai et al. 2024). The NLR reflects the balance and imbalance between neutrophils and lymphocytes in the blood (Hou et al. 2022). An increase in the NLR has been reported in cSVD patients and participants with cSVD markers (Hou et al. 2022; Jiang et al. 2022; Wang et al. 2022; Cai et al. 2024; Nam et al. 2024; Xiao et al. 2024). No difference was reported in participants with CMBs versus without (Nam et al. 2024).

Furthermore, two studies that investigated cSVD patients reported that the NLR was associated with WMH (Wang et al. 2022; Cai et al. 2024). More specifically, NLR was associated with periventricular WMH (PMWH) and deep WMH (DWMH) (Wang et al. 2022; Cai et al. 2024). These findings were partially supported by Nam et al*.,* who reported an association between NLR and WMH volume in healthy participants that were checked for cSVD imaging markers, however, they did not differentiate between PWMH and DWMH (Nam et al. 2017). In addition, the longitudinal study by Xiao et al*.* that included participants with cSVD markers reported that the NLR was associated with the progression of WMH volume (Xiao et al. 2024). In contrast, the NLR was not associated with WMH in two studies that included healthy participants with cSVD imaging markers (Chung et al. 2020; Jiang et al. 2022). However, in the study by Chung et al*.*, the prevalence of WMH was higher in the highest NLR group (Chung et al. 2020).

Furthermore, fives studies that included both cSVD patients and participants with markers of cSVD consistently reported no association between the NLR and lacunes (Chung et al. 2020; Jiang et al. 2022; Cai et al. 2024; Nam et al. 2024; Xiao et al. 2024).

Next, in a cohort study with cSVD patients, the NLR was related to ePVS (Wang et al. 2022). Similarly, the NLR was associated with moderate-to-severe BG-ePVS and ePVS in centrum semiovale (CSO-ePVS) in patients with cSVD (Cai et al. 2024). The NLR was not associated with severe BG-ePVS in participants with markers of cSVD (Jiang et al. 2022).

Finally, the NLR was not associated with CMBs in participants with cSVD markers (Jiang et al. 2022; Nam et al. 2024; Xiao et al. 2024). The association of the NLR with newer imaging markers was not reported.

Complementing the neuroimaging findings, Hou et al*.* further explored the relationship between the NLR and cognitive impairment in cSVD patients (Hou et al. 2022). They demonstrated that the NLR was positively correlated with cognitive impairment in cSVD patients (Hou et al. 2022).

In conclusion, the NLR showed mainly positive associations with WMH volume and ePVS in different brain regions, while no associations were observed with CMBs or lacunes in cSVD patients and individuals with cSVD imaging markers.

#### Lymphocyte-to-monocyte ratio

The lymphocyte-to-monocyte ratio (LMR) emerged as another potential inflammatory marker in cSVD, reflecting the balance between innate and adaptive immunity (Noz et al. 2021; Xiao et al. 2024). Li et al. reported lower LMR in cSVD patients compared to controls (Li et al. 2023). In individuals with imaging markers of cSVD, the LMR was decreased in individuals with higher WMH grades while no difference in the LMR was reported in the presence of CMBs or lacunes (Xiao et al. 2024).

As previously mentioned, higher monocyte counts correlated with higher WMH volume in participants with cSVD markers (Xiao et al. 2024). Interestingly, a greater LMR was associated with slower WMH volume progression but not with CMBs or lacunes in the same population (Xiao et al. 2024).

## Discussion

Circulating immune cells, capable of producing inflammatory markers, might play a crucial role in the development and progression of cSVD. This systematic review comprehensively examined immune cell populations and their associations with cSVD imaging markers in both diagnosed cSVD patients and healthy participants in whom cSVD imaging markers were assessed.

Total WBC count demonstrated inconsistent associations with conventional and new cSVD imaging markers, suggesting that WBC counts may not be a reliable indicator of cSVD’s progression. Different WBC subtypes play distinct roles in inflammation and therefore investigating individual cell populations could provide more detailed insights into the inflammatory processes associated with cSVD.

Monocytes emerged as critical players in the progression of cSVD. Monocyte subsets exhibit distinct functional properties. In particular, the intermediate monocyte subset demonstrates enhanced inflammatory features, including increased receptor expression, enhanced phagocytic capacity, and robust cytokine production (Noz et al. 2018). While results on the association of total monocyte count with conventional and new cSVD imaging markers were inconclusive, a positive association with intermediate monocytes and WMH was shown in individuals with markers of cSVD (Noz et al. 2018). Given the central role of monocytes in the inflammatory processes driving cSVD, the mechanisms linking monocyte function to disease progression should be further investigated. In this context, the MHR emerged as a promising inflammatory marker. The MHR showed significant associations with WMH volume, lacunes, and CMBs in participants with cSVD markers (Nam et al. 2024). Future research should focus on further elucidating the mechanisms by which monocyte subsets and their activation state contribute to disease progression.

Several studies reported that the ratio between different circulating immune cells is more strongly and consistently associated with markers of cSVD than individual blood cell counts (Nam et al. 2017, 2024; Jiang et al. 2022; Wang et al. 2022; Cai et al. 2024; Xiao et al. 2024). Although findings on neutrophil and lymphocyte count individually in relation to imaging markers in cSVD patients have been inconsistent, studies consistently reported positive associations between the NLR with WMH volume, as well as ePVS in different brain regions in cSVD patients (Wang et al. 2022; Cai et al. 2024). However, the NLR was not associated with lacunes in both cSVD patients and participants with markers of cSVD (Chung et al. 2020; Jiang et al. 2022; Cai et al. 2024; Nam et al. 2024; Xiao et al. 2024). Additionally, the LMR was associated with slower WMH volume progression in individuals with cSVD markers, suggesting a potential protective role in cerebrovascular pathology (Xiao et al. 2024).

Beyond imaging markers, the NLR demonstrated associations with cognitive impairment in cSVD patients, highlighting its potential as prognostic marker (Hou et al. 2022). This aligns with previous research suggesting that NLR, as a marker of the inflammatory response, may serve as a useful predictive marker for cSVD. It has previously been shown that individuals with mild cognitive impairment and early Alzheimer’s disease have an increased NLR compared to controls (Kalelioglu et al. 2017). Moreover, the NLR has been associated with an increased risk of cognitive impairment in elderly and in patients with acute ischemic stroke (Liu et al. 2020; Lee et al. 2021). Lastly, a high positive predictive effect of the NLR in the diagnosis and prognosis of many cardiovascular diseases has been shown (Afari and Bhat 2016). Taken together, these findings highlight the need for further research to elucidate the mechanisms underlying the associations between different immune cell ratios and cSVD imaging markers.

The variation in blood cell counts and associations with cSVD imaging markers found within and between studies highlights the complexity of the underlying immune mechanisms in cSVD. Multiple explanations exist for the variability in findings.

First, most studies employed retrospective, single-centered, cross-sectional study designs with small sample sizes, potentially leading to selection bias. These designs can suggest an association between circulating immune cells and cSVD but cannot guarantee causal relationships.

Second, the current research does not consider the dynamics of systemic inflammation as the associations of immune cells with cSVD were analyzed at one timepoint only. As cSVD is a chronic pathology, it may develop over many years. This may explain the unstable relationships between blood cell counts and cSVD progression. Longitudinal studies are needed to understand the dynamic inflammatory mechanisms that contribute to cSVD’s pathology.

Third, detailed immunophenotyping of cSVD patients is lacking. Characterization of immune cell populations, including their activation states, functional capabilities, and interactions, should be investigated in more detail to better understand the immune mechanisms driving cSVD pathogenesis.

Fourth, WMH seemed to be more often studied than the other imaging markers. Periventricular and deep WMH are known to have different pathological etiologies, as well as lobar and deep CMBs. By correlating specific immune cell populations with distinct cSVD imaging markers, the complex immunological mechanisms can be elucidated. Moreover, future studies should take into account novel imaging features such as DMVs, free water, and PSMD alongside conventional cSVD markers to provide a more comprehensive understanding of the disease (Duering et al. 2023).

Fifth, it is important to note the variation in study populations across the included studies. Half of the included studies investigated the relation between circulating immune cells and cSVD imaging markers in patients with established cSVD, typically using standardized imaging criteria such as STRIVE. The remaining studies focused on participants from community-based or general population cohorts who exhibited imaging markers of cSVD but lacked formal diagnosis or clinical symptoms. This methodological difference is important when interpreting results, however, given that the scope of this review was to assess circulating immune cells and markers of cSVD, studies including both cSVD patients and participants with imaging features of cSVD remain highly relevant. Understanding the associations in both study types is essential for identifying potential therapeutic targets, and distinguishing between them enhances clarity and applicability of findings in the broader context of cSVD research.

Lastly, immune cell counts can be affected by various underlying diseases or drugs. The impact of various comorbidities was not included in this review and could explain the inconsistency in results. To address these limitations, comprehensive, prospective studies are needed. Such studies should include detailed immunophenotyping, standardized neuroimaging protocols, and associations of specific immune cell populations with different cSVD manifestations. As such, immune cells may serve as biomarkers and/or therapeutic targets to slow or prevent cSVD progression. Considering the relative affordability and convenience of peripheral blood collection, circulating immune markers can be of interest in recognizing high-risk populations of cSVD.

## Conclusion

This systematic review shows that the relationship between circulating immune cells and markers of cSVD remains incompletely understood. Current evidence suggests a role for pro-inflammatory monocytes and derived ratios such as NLR, MHR, and LMR in the inflammatory processes underlying cSVD. These markers demonstrate potential as innovative diagnostic and prognostic targets. However, the results across studies are inconsistent, and further research is needed to clarify the exact relationship between these markers and disease progression.

## Supplementary Information

Below is the link to the electronic supplementary material.

Supplementary file1 (DOCX 101 KB)

## Data Availability

No datasets were generated or analysed during the current study.
