# Supplementary material for: Circulating immune cells in cerebral small vessel disease: a systematic review
Source: Biogerontology. 2025 May 5;26(3):101. doi: 10.1007/s10522-025-10250-x (PMC12052918; doi:10.1007/s10522-025-10250-x)
Supplement: Supplementary file 1 — Supplementary file1 (DOCX 101 KB) [file 10522_2025_10250_MOESM1_ESM.docx]

**Supplementary material**

**Circulating Immune Cells in Cerebral Small Vessel Disease: A Systematic Review**

L. Van der Taelen^1,3^, A.M. Briones^5^, T. Unger^3^, J. Staals^2,3^, R.J. van Oostenbrugge^2,3,4^, S. Foulquier^1,2,3,4^

^1^Dept of Pharmacology and Toxicology, Maastricht University, The Netherlands.

^2^Dept of Neurology, Maastricht University Medical Center, The Netherlands.

^3^CARIM – Research institute of Cardiovascular Diseases, Maastricht University, The Netherlands.

^4^MHeNS – Research institute of Mental Health and Neuroscience, Maastricht University, The Netherlands.

^5^Departamento de Farmacología, Facultad de Medicina, Universidad Autónoma de Madrid, Instituto de Investigación Hospital Universitario La Paz, Madrid, CiberCV, Spain.

**Corresponding author**

Dr Sébastien Foulquier, Pharm.D., Ph.D.

Maastricht University, Faculty of Health Medicine and Life Sciences

Department of Pharmacology and Toxicology

Universiteitssingel 50, 6229 ER Maastricht

P.O. Box 616, 6200 MD Maastricht, The Netherlands

[s.foulquier@maastrichtuniversity.nl](mailto:s.foulquier@maastrichtuniversity.nl)

**Acknowledgements**

None

**Funding**

This research was supported by the European Union’s Horizon 2020 research and innovation programme under the Marie Skłodowska-Curie (grant agreement No 954798).

**
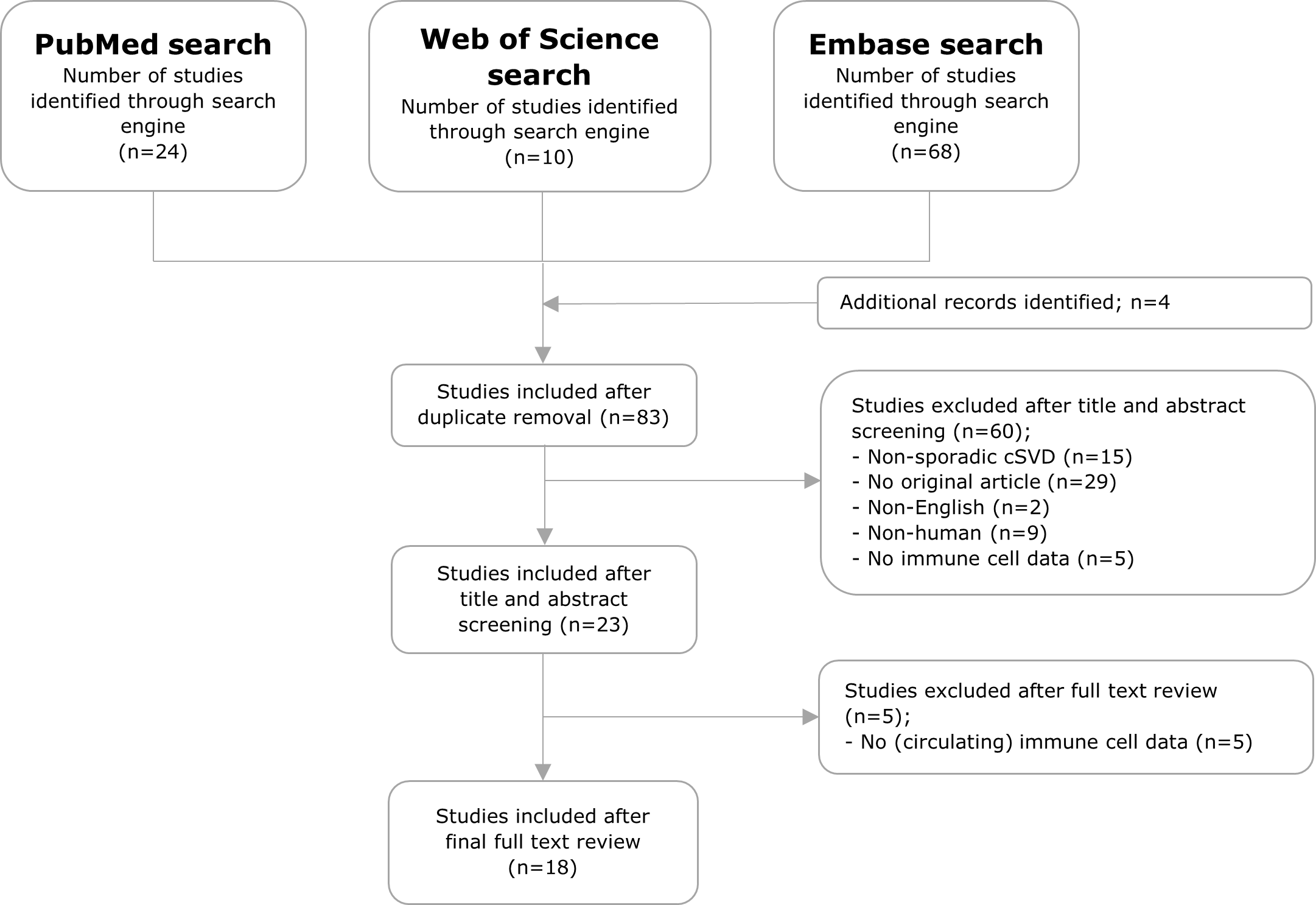
**

**Fig. S1** Flow diagram illustrating the systematic research protocol and the identification of the

corresponding publication record
